# Supplementary material for: Unveiling microbial succession dynamics on different plastic surfaces using WGCNA
Source: PLoS One. 2025 Feb 6;20(2):e0318843. doi: 10.1371/journal.pone.0318843 (PMC11801547; doi:10.1371/journal.pone.0318843)
Supplement: S1 File — (DOCX) [file pone.0318843.s001.docx]

**Unveiling microbial succession dynamics on different plastic surfaces using WGCNA**

Keren Davidov^1^, Sheli Itzahri^1^, Liat Anabel Sinberger^1^ & Matan Oren^1*^

1. Department of Molecular Biology, Ariel University, Ariel, Israel.

* Correspondence to: [matanor@ariel.ac.il](mailto:matanok@ariel.ac.il)

**Supplementary Figures**


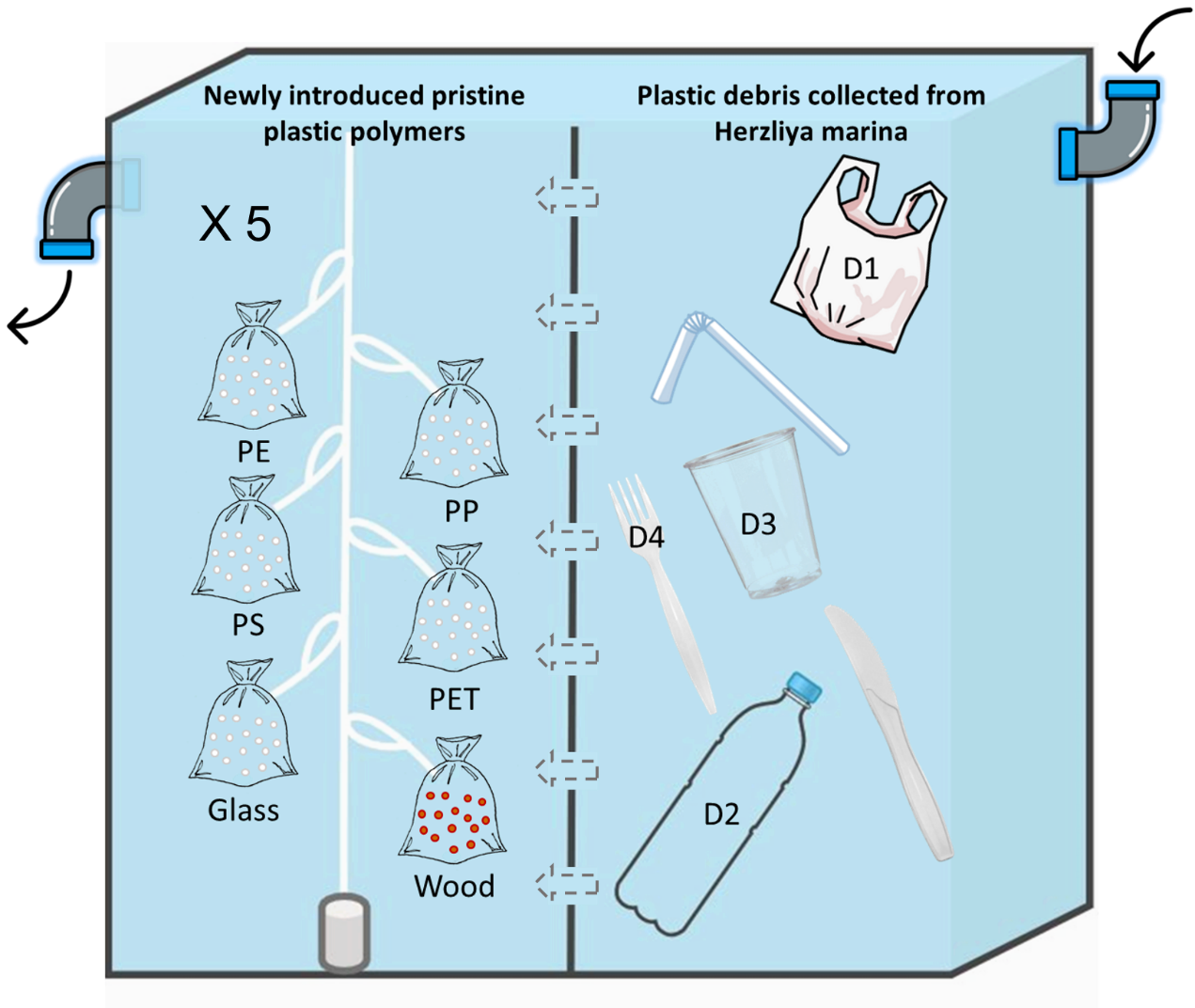


**Supplementary figure S1. Experiment setup.** The two aquarium compartments were separated by a polycarbonate barrier with round holes, 2 cm in diameter, 7.5 cm apart, enabling water interchange (dashed line). On the left compartment – organza mesh bags containing beads made of different plastic polymers, wood and glass (5 replicates of each of 6 materials). On the right compartment – plastic items collected from the environment (represented by D1-D4 according to the items that were sampled). Water inflow is to the aquarium far right corner; water outflow is from the far-left corner. The second experiment included only PE, PET, glass and wood on the left compartment. Sump included physical filtration of particles and a protein breakdown appartatus (skimmer).


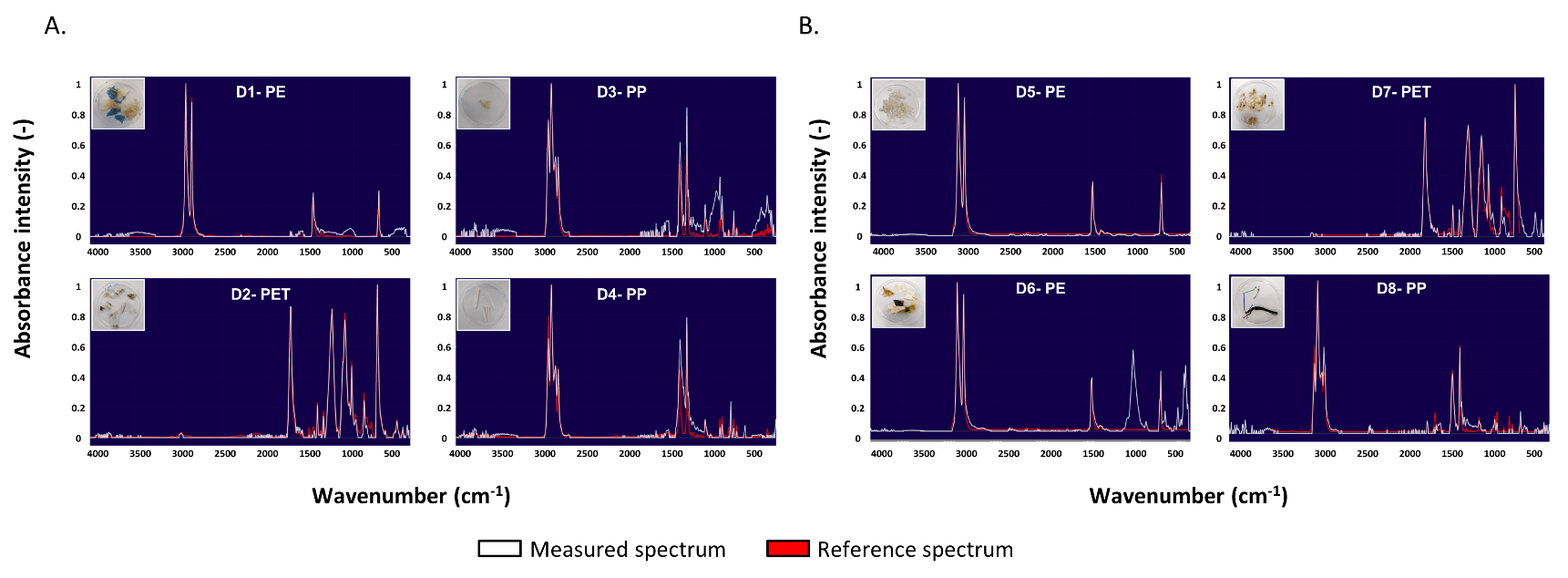


**Supplementary figure S2. FTIR spectral analysis of plastic debris.** A. Plastic debris items for the main experiment (D1-D4). B. Plastic debris items for the second experiment (D5-D8). The measured spectra are in white and the reference spectra are in red.


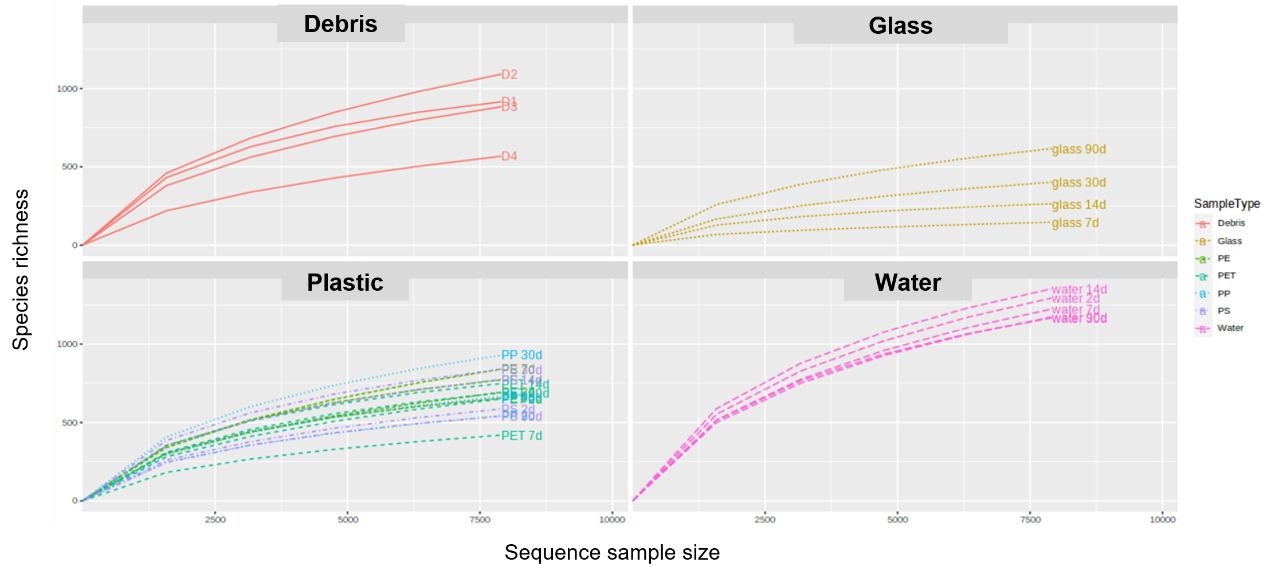
**Supplementary figure S3. Biodiversity rarefication curves** A. Rarefication curves for Debris, Glass, Plastic and Water samples – species richness by sample size. B. Alpha Diversity (Chao1 and Shannon indexes) across all sample groups (Debris, Glass, PE, PP, PET, PS and Water).


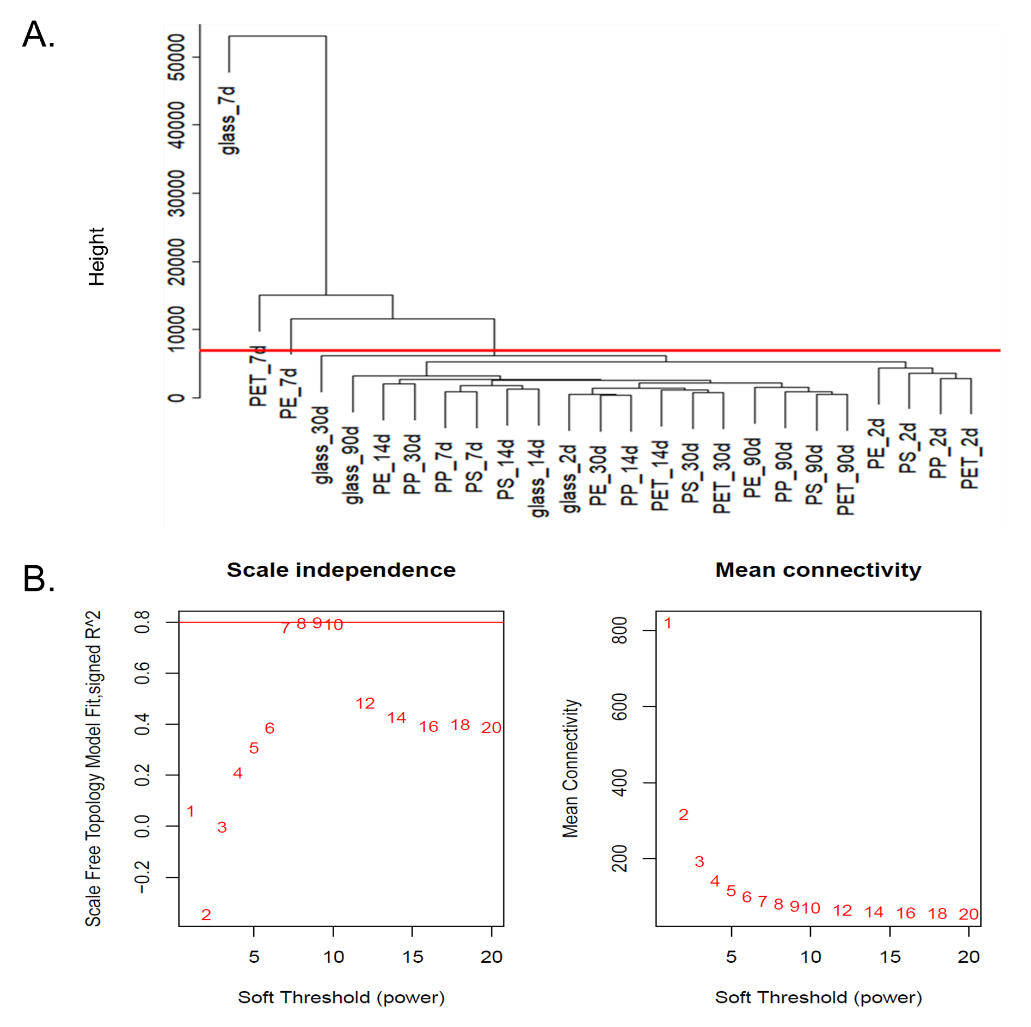


C.

**Supplementary figure S4. Data pre-processing for WGCNA**. A. Sample dendrogram and trait heatmap after removing the outlying samples. The dendrogram illustrates the hierarchical clustering of samples. Glass, PET, and PE 7 days were removed from the analysis. B. Scale Independence plot presents the scale-free topology fit index as a function of the soft-thresholding power. The red horizontal line represents the cutoff (R^2 = 0.80) for optimal scale independence. C. Mean connectivity plot presents the mean connectivity of the network as a function of the soft-thresholding power. The red labels indicate the corresponding powers used for analysis.


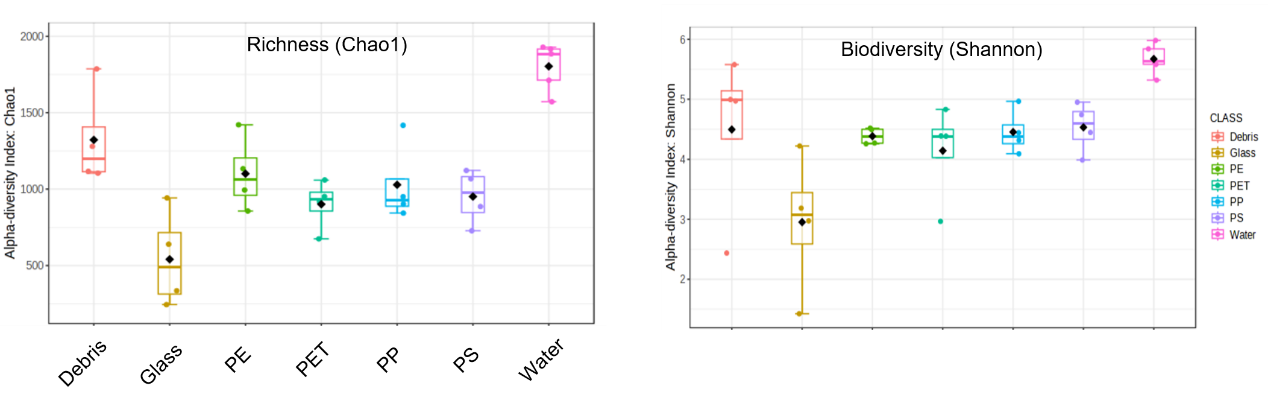


**Supplementary figure S5. Biodiversity indexes for all surfaces.**

**S1 Table. General run parameters – Experiment 1**

| **Sample** | | **Total number of reads** | **Mapped reads (LCA==0)** | **% mapped reads** | **No. of species** | **MinION flow cell** |
| --- | --- | --- | --- | --- | --- | --- |
| **2 days** | PE | 72,968 | 25,122 | 34.43 | 710 | **1** |
|  | PP | 90,944 | 31,581 | 34.73 | 601 | **3** |
|  | PS | 44,689 | 23,086 | 51.66 | 587 | **1** |
|  | PET | 76,430 | 26,626 | 34.84 | 702 | **1** |
|  | Glass | 2,277 | 1,337 | 58.72 | 79 | **3** |
|  | Wood | 518 | 305 | 58.88 | 27 | **1** |
|  | Water | 87,178 | 22,406 | 25.70 | 1,329 | **1** |
| **7 days** | PE | 157,897 | 43,933 | 27.82 | 1,157 | **1** |
|  | PP | 20,736 | 8,294 | 40.00 | 391 | **1** |
|  | PS | 13,319 | 7,336 | 55.08 | 299 | **1** |
|  | PET | 51,809 | 35,664 | 68.84 | 530 | **3** |
|  | Glass | 78,470 | 73,857 | 94.12 | 245 | **1** |
|  | Wood | 7,565 | 5,001 | 66.11 | 145 | **1** |
|  | Water | 61,875 | 18,657 | 30.15 | 1,126 | **1** |
| **14 days** | PE | 42,917 | 9,425 | 21.96 | 503 | **2** |
|  | PP | 16,711 | 3,395 | 20.32 | 331 | **2** |
|  | PS | 46,572 | 13,037 | 27.99 | 591 | **3** |
|  | PET | 33,924 | 9,006 | 26.55 | 487 | **3** |
|  | Glass | 11,132 | 8,487 | 76.24 | 167 | **3** |
|  | Wood | 184 | 79 | 42.93 | 16 | **3** |
|  | Water | 60,091 | 13,667 | 22.74 | 1,105 | **2** |
| **30 days** | PE | 11,923 | 2,119 | 17.77 | 195 | **2** |
|  | PP | 73,689 | 1,4833 | 20.13 | 773 | **2** |
|  | PS | 57,996 | 11,460 | 19.76 | 613 | **3** |
|  | PET | 28,734 | 5,374 | 18.70 | 260 | **3** |
|  | Glass | 41,877 | 20,539 | 49.05 | 369 | **3** |
|  | Wood | 25,350 | 9,455 | 37.30 | 309 | **3** |
|  | Water | 38,608 | 10,380 | 26.89 | 818 | **2** |
| **90 days** | PE | 62,930 | 9,483 | 15.07 | 436 | **3** |
|  | PP | 42,039 | 10,364 | 24.65 | 437 | **3** |
|  | PS | 43,490 | 9,751 | 22.42 | 340 | **3** |
|  | PET | 60,616 | 12,099 | 19.96 | 482 | **3** |
|  | Glass | 43,357 | 17,441 | 40.23 | 555 | **3** |
|  | Wood | 11,253 | 2,662 | 23.66 | 204 | **3** |
|  | Water | 64,132 | 18,742 | 29.22 | 1,138 | **3** |
| **Debris** | D1 | 29,231 | 7,940 | 27.16 | 556 | **1** |
|  | D2 | 265,893 | 65,849 | 24.77 | 1,963 | **1** |
|  | D3 | 71,037 | 17,864 | 25.15 | 761 | **1** |
|  | D4 | 209,368 | 119,008 | 56.84 | 1,277 | **1** |

| **Sample** | | **Total number of reads** | **Mapped reads (LCA==0)** | **% mapped reads** | **No. of species** | **MinION flow cell** |
| --- | --- | --- | --- | --- | --- | --- |
| **7 days** | PE | 189,159 | 48,159 | 25.46 | 1,133 | **4** |
|  | PET | 3,880 | 1,656 | 42.68 | 150 |  |
|  | Glass | 910 | 586 | 64.40 | 48 |  |
|  | Wood | 407,636 | 287,567 | 70.55 | 1,078 |  |
|  | Water | 44,870 | 14,261 | 31.78 | 836 |  |
| **14 days** | PE | 194,577 | 37,580 | 19.31 | 901 |  |
|  | PET | 52,028 | 12,728 | 24.46 | 746 |  |
|  | Glass | 688 | 238 | 34.59 | 28 |  |
|  | Wood | 74,247 | 52,727 | 71.02 | 492 |  |
|  | Water | 14,2815 | 36,235 | 25.37 | 1,666 |  |
| **30 days** | PE | 183,080 | 30,897 | 16.88 | 1,189 |  |
|  | PET | 3,201 | 597 | 18.65 | 102 |  |
|  | Glass | 1,180 | 361 | 30.59 | 47 |  |
|  | Wood | 195 | 80 | 41.03 | 14 |  |
|  | Water | 120,956 | 25,272 | 20.89 | 1,441 |  |
| **Debris** | D1 | 3,833 | 706 | 18.42 | 69 |  |
|  | D2 | 17,115 | 4,166 | 24.34 | 312 |  |
|  | D3 | 2,112 | 431 | 20.41 | 69 |  |
|  | D4 | 76,264 | 18,296 | 23.99 | 984 |  |

**S2 Table. General run parameters – Experiment 2**

| **PET** | **PS** | **PP** | **PE** |
| --- | --- | --- | --- |
| *Catenovulum agarivorans* (0.37) *Desulfomicrobium macestii* (0.11%)  *Kordiimonas lacus* (0.18%)  *Methyloversatilis discipulorum* (0.15%)  *Pelagicoccus croceus* (0.18%)  *Ruegeria arenilitoris* (0.2%)  *Ruegeria atlantica* (0.1%)  *Ruegeria conchae* (0.34%) | *Granulosicoccus antarcticus* (0.44%)  *Granulosicoccus coccoides* (0.95%)  *Kangiella profundi* (0.15%)  *Marinobacter salinus* (0.19%)  *Oceanibaculum pacificum* (0.11%)  *Paraburkholderia dinghuensis* (0.1%)  *Paraburkholderia nodosa* (0.11%)  *Pelomonas aquatica* (0.14%)  *Pelomonas saccharophila* (2.9%)  *Pseudomonas entomophila* (0.28%)  *Rickettsia bellii* (0.14%)  *Thermogutta terrifontis* (0.25%) | *Actinomarinicola tropica* (0.14%)  *Alcanivorax borkumensis* (0.11%)  *Alcanivorax dieselolei* (3.1%)  *Alcanivorax hongdengensis* (3.43%)  *Alcanivorax marinus* (0.13%)  *Alcanivorax pacificus* (0.32%)  *Geobacter luticola* (0.25%)  *Geobacter pelophilus* (0.17%)  *Halioglobus japonicus* (0.23%)  *Halioglobus lutimaris* (0.45%)  *Marinobacterium stanieri* (0.13%)  *Nevskia persephonica* (0.14%)  *Nevskia ramosa* (0.16%)  *Pseudomonas matsuisoli* (0.11%)  *Pseudomonas protegens* (0.21%)  *Salinisphaera aquimarina* (0.91%)  *Salinisphaera dokdonensis* (0.29%)  *Salinisphaera shabanensis* (0.1%)  *Sandaracinus amylolyticus* (0.13%)  *Sneathiella glossodoripedis* (1.61%)  *Tepidicaulis marinus* (0.32%) | *Aestuariispira insulae* (0.11%)  *Alcanivorax nanhaiticus* (15.3%)  *Alcanivorax jadensis* (0.42%)  *Amphritea japonica* (0.12%)  *Bdellovibrio bacteriovorus* (0.14)  *Ketobacter alkanivorans* (6.11)  *Marinicella litoralis* (1.11%)  *Marinobacterium mangrovicola* (0.18%)  *Marinobacterium zhoushanense* (0.11%)  *Microbulbifer variabilis* (0.48%)  *Microbulbifer thermotolerans* (0.39%)  *Microbulbifer gwangyangensis* (0.12%)  *Peredibacter starrii* (0.21%)  *Porticoccus litoralis* (5.35%)  *Pseudomonas reidholzensis* (0.3%)  *Pseudomonas jilinensis* (0.27%)  *Pseudomonas straminea* (0.2%)  *Pseudomonas mucidolens* (0.16%)  *Pseudomonas parafulva* (0.14%)  *Pseudomonas monteilii* (0.13%)  *Spongiibacter marinus* (1.65%)  *Umboniibacter marinipuniceus* (0.11%) |

**S3 Table . Significantly enriched species on plastic polymers ***

* Significantly enriched species on plastic polymers. Numbers in brackets indicate the average relative abundance within one month incubation time.
