# Supplementary material for: Unveiling microbial succession dynamics on different plastic surfaces using WGCNA
Source: PLoS One. 2025 Feb 6;20(2):e0318843. doi: 10.1371/journal.pone.0318843 (PMC11801547; doi:10.1371/journal.pone.0318843)
Supplement: Attachment — Submitted filename: Response to Reviewers plosone final_2.docx [file pone.0318843.s003.docx]

**Dear Dr. Arga Chandrashekar Anil,**

**Academic Editor, PLOS ONE.**

We thank you for providing us with the opportunity to revise our manuscript, entitled "Unveiling microbial succession dynamics on different plastic surfaces using WGCNA" We are grateful for the feedback and have carefully addressed all points raised during this second round of review. Below, we provide detailed responses to the comments and outline the corresponding revisions made to the manuscript.

**Editor Comments:**

1. I have one minor point to be made regarding point 12 of the comments offered by reviewer 1: the authors can include a statement to manuscript and not simply brush aside the obvious.

**Response:** This comment refers to reviewer 1 comments: "No mention of extraction or PCR blanks in methods. This is highly problematic for publication and must be addressed before the paper can be published."

In our initial response, we explained that PCR blanks are routinely used in all our PCR workflows to test for primer and foreign DNA contamination and indicated that we did not think it was necessary to explicitly state this in the manuscript. However, we now understand the importance of providing this information explicitly to ensure transparency and to align with the journal’s expectations. Accordingly, we have revised the Methods section to explicitly mention the inclusion of PCR blanks. The updated text now states: “All PCR procedures include PCR blanks to detect potential foreign DNA contamination in the water, reagents or primers" (line 166). We believe this addition addresses the concern and provides the necessary detail to ensure clarity for readers.

**Journal Requirements:**

2. Please review your reference list to ensure that it is complete and correct. If you have cited papers that have been retracted, please include the rationale for doing so in the manuscript text, or remove these references and replace them with relevant current references. Any changes to the reference list should be mentioned in the rebuttal letter that accompanies your revised manuscript.

**Response:** We have thoroughly reviewed the reference list to ensure that all citations are accurate and complete.

We trust that these revisions and responses address all concerns raised by the reviewers and the editorial team. We appreciate your continued guidance throughout this process and are happy to address any further questions or requirements.

Thank you for your time and consideration.

For the Authors,

Keren Davidov & Matan Oren
